# Falls Risk and Simulated Driving Performance in Older Adults

**DOI:** 10.1155/2013/356948

**Published:** 2013-02-21

**Authors:** John G. Gaspar, Mark B. Neider, Arthur F. Kramer

**Affiliations:** ^1^Department of Psychology and Beckman Institute for Advanced Science and Technology, University of Illinois at Urbana-Champaign, Urbana, IL 61801, USA; ^2^Department of Psychology, University of Central Florida, Orlando, FL 32816, USA

## Abstract

Declines in executive function and dual-task performance have been related to falls in older adults, and recent research suggests that older adults at risk for falls also show impairments on real-world tasks, such as crossing a street. The present study examined whether falls risk was associated with driving performance in a high-fidelity simulator. Participants were classified as high or low falls risk using the Physiological Profile Assessment and completed a number of challenging simulated driving assessments in which they responded quickly to unexpected events. High falls risk drivers had slower response times (~2.1 seconds) to unexpected events compared to low falls risk drivers (~1.7 seconds). Furthermore, when asked to perform a concurrent cognitive task while driving, high falls risk drivers showed greater costs to secondary task performance than did low falls risk drivers, and low falls risk older adults also outperformed high falls risk older adults on a computer-based measure of dual-task performance. Our results suggest that attentional differences between high and low falls risk older adults extend to simulated driving performance.

## 1. Introduction

Per mile driven, adults over age 65 are more likely to be involved in motor vehicle collisions than are younger experienced drivers [1], and declines in attention are related to older driver impairment [2, 3]. Attention is also critical to balance and gait, especially for older adults. Walkers must monitor for changes in environment and plan their next step. When a walking or balance task is combined with a cognitively challenging secondary task (e.g., memorizing a list of words), performance decrements are found for both tasks relative to performing each task separately [4]. These dual-task costs suggest that walking competes for shared attentional resources, as predicted by resource models of attention [5–9]. 

Older adults often have increased difficulty when multitasking, including paradigms that involve balancing or walking [10–12]. For example, older adults show larger dual-task costs on walking while memorizing task than do younger adults [13–16]. Such declines in multitasking ability are theorized to result in an increased risk for falls among older adults. Approximately 30% of community-dwelling older adults experience one or more falls annually [17, 18]. Age-related declines in the ability to multitask are related to an increase in falls risk. For example, performance on a counting while walking task predicts falls in older adults ([19]; see also [20–22]). Similarly, older adults at high risk for falls are less successful than low falls risk adults when crossing the street in a simulated environment while talking on a hands-free cell phone [23]. 

Differences in multitasking ability that have been associated with falls risk are theorized to result from declines in executive control, the functions which select, schedule, and coordinate task processes. Low falls risk older adults outperform high falls risk adults on tasks theorized to index executive control abilities [24–26]. Further, measures of executive control predict laboratory dual-task performance [27]. This suggests that older adults with poorer executive control are worse at managing complex task demands pertaining to balance or gait and are, therefore, more likely to fall.

Executive control is also important for other real-world tasks, such as driving. Drivers must attend to several areas of the environment and plan and execute responses to avoid collisions. Indeed, poorer performance on executive control tasks is predictive of retrospective crashes in a sample of older male drivers [28], and models of crash risk often comprise multidisciplinary factors, including physical ability, attention, and health [29–31]. Importantly, data also suggest a link between falls risk and driving. A history of falls is associated with older driver crashes [32], and some models of crash risk actually incorporate falls risk among other physical and cognitive measures [33]. Importantly, however, the results linking falls risk and driving performance rely on crash reports and subjectively rated driving performance and do not identify the behaviors related to unsafe driving in high falls risk older adults. 

The goal of the present study was to explore the relationship between falls risk and driving in older adults in greater detail using a high-fidelity driving simulator, which allowed us to place drivers in potentially dangerous situations and to collect objective performance measures. We also included a battery of cognitive tasks to examine the relationship between falls risk, cognition, and simulated driving. Given previous findings of heightened crash risk, we predicted that low falls risk drivers would outperform high falls risk drivers on our simulator driving assessments. We further predicted that high falls risk drivers would show the greatest performance decrements in simulated driving performance under high multitasking load (i.e., when responding to unexpected events). Finally, we predicted that low falls risk older adults would outperform high falls risk adults on a desktop computer dual-task paradigm.

## 2. Methods

### 2.1. Participants

36 independent-living older adults were recruited from the Urbana-Champaign community and paid $8 per hour for participating. All participants demonstrated normal or corrected-to-normal visual acuity (20/30 or better using a Snellen chart) and normal color vision (Ishihara Color Vision Test) and scored above 28 (of 30) on the Folstein minimental state exam. All participants had valid drivers' licenses and drove regularly. Mobility and balance were assessed using the Timed Up and Go test (TUAG) [34]. Descriptive data are provided in Table 1.

### 2.2. Apparatus and Falls Risk Assessment

The Beckman Institute Driving Simulator at the University of Illinois (http://isl.beckman.illinois.edu/) was used to assess simulated driving performance. The simulator consists of a General Motors Saturn automobile surrounded by eight screens. Traffic environments and experimental scenarios were developed using HyperDrive Authoring Suite. Data was recorded at 60 Hz. A PC with a 19-inch screen was used for neuropsychological testing. All tasks were programmed using E-prime (Psychology Software Tools). Viewing distance was approximately 77 cm for all tasks, although participants were free to move their heads. 

#### 2.2.1. Falls Risk

Participants completed a falls history questionnaire (i.e., “have you fallen in the last 6 months?” “how many times?”). Only three individuals reported falling in the previous 6 months. Thus, we classified participants as high or low falls risk based on scores from the Physiological Profile Assessment (PPA), as described by Lord and colleagues [35]. The PPA is a composite falls risk score based on measures of edge contrast sensitivity, hand reaction time, proprioception, leg muscle strength, and sway, which have shown to reliably predict falls in community and institutional settings [36, 37]. We set an a priori cutoff score of 0.6 to classify high and low falls risk (i.e., high falls risk ≥ 0.6) [38]. Fifteen participants were classified as high falls risk (mean age = 75.8, age range = 71–80), and 15 participants were classified as low falls risk (mean age = 74.4, age range = 67–80). The high and low falls risk groups were statistically similar in age, driving experience, and current driving habits. PPA scores were significantly correlated with times on the TUAG test (*r* = .61, *P* = .001).

### 2.3. Cognitive Battery

#### 2.3.1. Computer Dual Task

Participants performed two tasks both separately and simultaneously. For one task, participants determined whether a letter was an A or B and pressed corresponding keys with their right hand. In the second task, participants determined whether a number was a 2 or 3 and pressed a corresponding key with their left hand. On single-task trials (50%), participants performed only one task. On dual-task trials (50%), they performed both tasks. The primary performance measure was reaction time. Participants completed single-task and dual-task practice trials, followed by a block of forty intermixed single- and dual-task test trials. 

#### 2.3.2. Functional Field of View (FFOV)

Participants searched for a white triangle within a circle among square distracters in a briefly (44 ms) presented display. Targets were presented with equal probability on one of 8 radial spokes at eccentricities of 10°, 20°, and 30° from fixation. The search display was followed by a 100 ms mask consisting of random black and white lines and shapes. Participants then clicked with the mouse on the spoke where the target appeared. The percentage of targets correctly localized was the critical measure of performance. This task is similar to the peripheral localization subtask of the Useful Field of View [3], and we theorized that this measure might be predictive of older drivers' ability to respond to peripheral events. Participants completed 24 practice trials followed by 120 test trials. 

#### 2.3.3. Realistic Change Detection

Participants performed a flicker change detection task [37]. Stimuli were 80 pairs of photographs of real driving scenes taken from the driver's perspective. Each pair of images differed in one detail (e.g., a car in one image was removed from the other image). On each trial, participants saw a repeating cycle of 4 images, first image (240 ms), a gray mask screen (80 ms), the modified image (240 ms), and a gray mask screen (80 ms), and pressed a key when they detected the change. The screen froze, and participants clicked on the change location with the mouse. Reaction time and accuracy were used as performance measures. Participants had 30 seconds to respond and completed 1 practice trial followed by 40 test trials. 

### 2.4. Simulator Driving Assessment

#### 2.4.1. Following Task

Drivers followed a lead vehicle (LV) along a straight, two-lane highway for approximately 15 minutes. Participants were instructed to maintain a 5-second gap from the LV, which traveled at 45 mph. During the practice drive, participants received auditory feedback to help visualize the 5-second gap. At 20 random times during the test drives, the LV's brake lights illuminated and its speed decreased. Drivers were instructed to brake as soon they detected LV slowing. When the driver pressed the brake, the LV accelerated back to 45 mph. Performance measures included response time to LV braking, following distance, and lane keeping.

#### 2.4.2. Hazard Task

**Figure 1:**
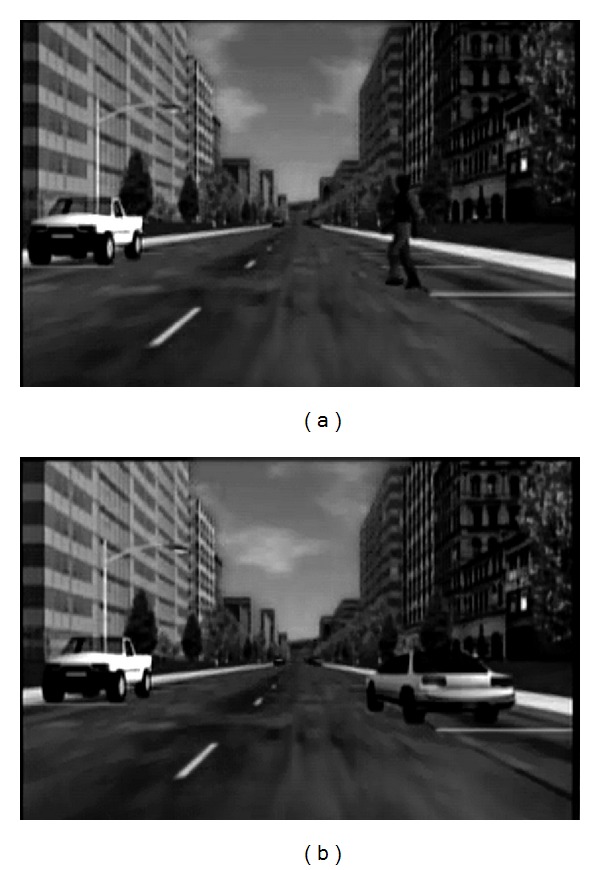
Examples of potential hazards in the hazard driving task. In (a), a pedestrian crosses the street in front of the driver. In (b), a parked vehicle starts to pull out in front of the driver.

Drivers responded to potentially hazardous events as they drove along a straight, two-lane urban road for approximately 15 minutes. Ambient traffic and pedestrians were randomly generated such that there was a constant stream of traffic in the opposite lane, and the sidewalks were crowded with pedestrians. Participants were instructed to maintain a speed of 35 mph. There were a total of 20 randomly spaced potential hazards in each drive. Hazards comprised pedestrians crossing the roadway and cars on the right shoulder beginning to pull out and stopping (Figure 1). Participants were instructed to press the brakes as soon as detecting a hazard. Performance measures included brake response time and lane keeping.

#### 2.4.3. Secondary Task

Participants completed two versions of two separate simulated driving tasks. The order of the task conditions was counterbalanced across participants. In the drive-only condition, participants drove without secondary task distraction. In the drive + 1-Back condition, participants performed cognitively demanding secondary task, a continuous 1-Back task where they heard a letter every 3 seconds and indicated whether the letter was the same as or different from the previous letter via buttons on the steering wheel, while driving. Accuracy was considered the primary measure of secondary task performance.

### 2.5. Procedure

Participants completed three 1.5-hour sessions. Session 1 consisted of a screening drive for simulator sickness, descriptive measures, and falls risk assessment (6 potential participants showed signs of simulator sickness and were not included in the study). In sessions 2 and 3, participants completed the three computer-based cognitive tasks, followed by practice with the secondary task and two driving assessments in the simulator. The order of the cognitive tasks and driving task conditions was counterbalanced across subjects.

## 3. Results

### 3.1. Cognitive Battery

Three participants (2 high falls risk and 1 low falls risk) did not complete the cognitive battery (due to technical issues) and were not included in analyses of the cognitive tasks. Dual-task cost was calculated by subtracting the single-task reaction time from the dual-task reaction time. High falls risk participants had a significantly higher dual-task cost compared to the low falls risk group, *F*(1,23) = 6.88, *P* < .05. Single-task reaction times on the computer dual-task paradigm did not differ between the groups, *F*(1,23) = .19, *P* = .67, indicating that differences were not due to general slowing. Localization accuracy on the FFOV task did not differ between the falls risk groups (*P* > .70), nor did reaction time or accuracy on the change detection task (*P*'s > .35; see Table 1). 

### 3.2. Driving Assessment

Analyses were performed separately for each driving task. Driving measures were entered into an ANOVA with falls risk group (high versus low) as a between-subjects factor and task condition (drive-only versus drive + 1-Back) as a within-subjects factor. Two participants (1 high falls risk and 1 low falls risk) who had passed the screening drive showed signs of simulator sickness during the experimental drives, did not complete the study, and were excluded from analyses.

**Figure 2:**
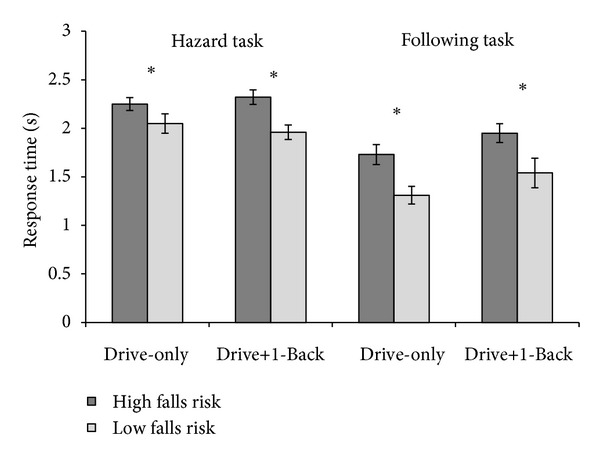
Brake response times. Mean brake response time in seconds for the high and low falls risk driver groups in drive-only and drive + 1-Back task conditions in the hazard response and car following paradigms. Error bars represent one standard error of the mean. *P < .05.

Collisions were infrequent in both driving tasks (Table 1), precluding statistical analysis. Response time (RT) was defined as the time it took a driver to press the brake pedal following the onset of the LV brake lights or the triggering of a hazard event (Figure 2). Only events where the driver avoided a collision were included in the analyses. Low falls risk drivers responded significantly faster than high falls risk drivers to LV braking events, *F*(1,26) = 11.28, *P* < .01. Low falls risk drivers also responded faster than high falls risk drivers to the onset of hazard events, *F*(1,26) = 9.32, *P* < .01. In the following task, performing the auditory 1-Back task slowed responses, *F*(1,26) = 5.24, *P* < .05, though this was not the case for the hazard drive, *F*(1,26) = .083, *P* = .78. 

We ran separate analyses using hand reaction time, contrast sensitivity, and the combination of hand reaction time and contrast sensitivity as covariates to investigate the impact of specific components of the PPA [38]. With hand reaction time as a covariate, low falls risk drivers still had significantly faster RTs than did high falls risk drivers (*P*'s < .05). When contrast sensitivity was included as a covariate, low falls risk drivers responded faster than did high falls risk drivers in the hazard (*F*(1,26) = 4.90, *P* < .05) but not the following (*F*(1,26) = 1.21, *P* > .10) simulated driving tasks. When both hand reaction time and contrast sensitivity were included as covariates, brake RT differences between groups were no longer significant (*P*'s > .10). 

High and low falls risk drivers did not differ in average velocity or lane keeping performance (all *P*'s > .10). On the following task, headway distance was defined as the average distance between the driver's vehicle and the LV. Drivers increased their headway in the drive + 1-Back condition, *F*(1,26) = 4.078, *P* = .05. However, performing the concurrent secondary task did not differentially impair high falls risk drivers (*P* > .40).

#### 3.2.1. Does Computer Dual-Task Performance Predict Driving Performance?

**Figure 3:**
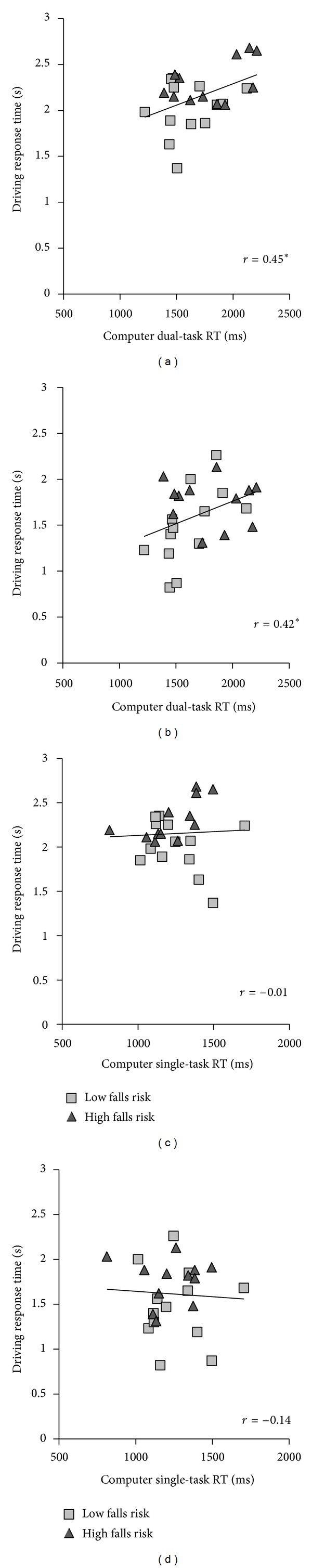
Driving response times and dual-task performance. Response time in seconds in the hazard (a) and (c) and following (b) and (d) driving tasks plotted against single-task and dual-task reaction time in milliseconds on the computer dual-task paradigm. *P < .05.

To examine whether performance on the computer dual-task paradigm predicted simulated driving, we computed the correlation between dual-task cost on the computer paradigm and RT in the driving tasks. Participants with a lower computer dual-task cost responded faster to both LV braking events (*r* = .42, *P* < .05) and to hazard events (*r* = .45, *P* < .05). Conversely, single-task performance in the computer dual-task paradigm was not correlated with RT in the simulated driving assessments (*P*'s > .20; Figures 3(c) and 3(d)).

#### 3.2.2. Secondary Task Performance

**Figure 4:**
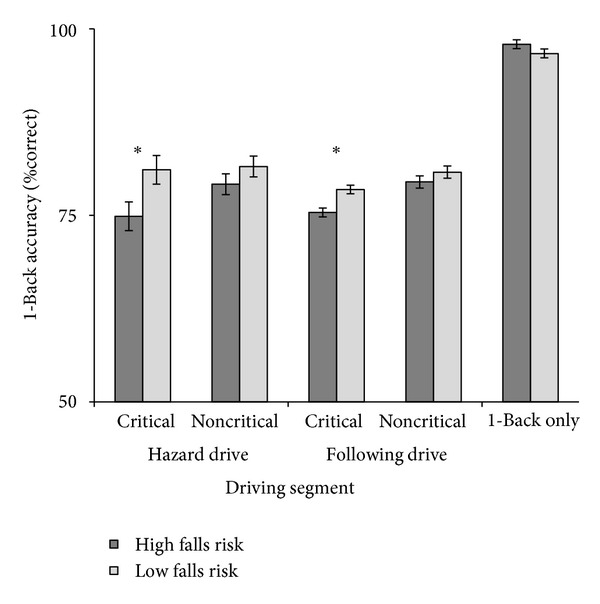
1-Back accuracy. Accuracy on the 1-Back task in critical and noncritical segments of the hazard and following drives and in the single-task (1-Back only) condition. Error bars represent one standard error of the mean. *P < .05.

We compared accuracy on the auditory 1-Back task during 1-Back only (during the last half of practice) and 1-Back + driving performances to examine whether there were costs to secondary task performance when driving (Figure 4) [39]. There was a significant cost to 1-Back accuracy in both the following drive (*F*(1,26) = 254.5, *P* < .001) and the hazard drive (*F*(1,26) = 173.0, *P* < .001), though there was no difference between falls risk groups (*P* > .15). To determine if a group difference existed when the driving task was most demanding, we divided 1-Back accuracy into critical segments (i.e., during peripheral hazard or LV braking events) and noncritical segments (i.e., periods between critical events). High falls risk participants were marginally worse than were low falls risk participants during critical periods in both the following (*F*(1,26) = 3.27, *P* = .084) and the hazard drives (*F*(1,26) = 3.57, *P* = .071). There were no differences in 1-Back accuracy between groups in the noncritical segments for either drive (*P*'s > .70; see Figure 3). This indicates that, when responding to critical events, high falls risk drivers showed larger costs to secondary task performance than did low falls risk drivers. Though this may have been a compensatory strategy, it did not eliminate group differences in RT to critical driving events.

## 4. Discussion

The current study compared the driving performance of high and low falls risk older adults in a high-fidelity driving simulator. Of greatest importance is the finding that high falls risk drivers responded approximately 400 ms slower than did low falls risk drivers to critical events. High falls risk drivers responded slower than did low falls risk drivers to both central lead vehicle braking events and to peripheral hazards. Such slower responses may be a contributing factor to heightened crash rates for high falls risk older adults reported elsewhere [32]. Indeed, the ability to detect hazards has been linked to crash involvement [40]. 

 Our data extend the literature that examines multitasking performance in older adults at high and low risk for falls [19–23]. In our cognitive battery, high falls risk participants had greater dual-task costs on the computer paradigm than did low falls risk older adults, and, importantly, this was not due to differences in general slowing. In much the same way as walking, responding to critical events while driving requires the ability to multitask; drivers must scan the environment and plan and execute evasive responses while controlling the vehicle. In our study, the ability to efficiently allocate attention among different tasks was most critical to responding to unexpected driving events in the simulator. This is supported by the finding that performance on the computer dual-task paradigm predicted driving RTs and further suggests that these multitasking differences between high and low falls risk older adults are somewhat general in nature. Previous research suggests that deficits in executive function likely underlie declines in multitasking performance and mediate the relationship between balance and falls [24, 25, 27], as well as crash risk for older drivers [28]. Changes in executive control likely contribute to the general multitasking differences shown in our cognitive battery and simulator driving assessments.

Our results indicate that contrast sensitivity and response time were the most important components of the PPA relating to simulated driving RT. Previous work has found that contrast sensitivity and response time are important abilities in responding to driving hazards [41]. The present data suggest that these abilities are important to both walking and simulated driving.

We failed to find differences between high and low falls risk drivers on other simulator driving performance measures such as lane keeping. Hazard responses posed the highest multitasking demand in our driving assessments. Previous research has shown that multitasking differences in older adults and differences between high and low falls risk older adults arise primarily at the highest levels of task demand [13, 16, 23]. Thus, in the present study, high and low falls risk drivers performed equally well during relatively low-demand driving intervals, but high falls risk drivers were more impaired in high-demand situations, resulting in slower responses. In the driving + 1-Back condition, dual-task costs were found primarily in 1-Back accuracy. This may reflect a strategy whereby older adults compensated for higher task demands by sacrificing performance on the less safety-critical task [14]. During responses to critical events, high falls risk drivers sacrificed 1-Back accuracy more than did low falls risk drivers, which again suggests high falls risk participants struggled under high multitasking demand.

Future work should explore the contribution of different components of executive control (e.g., switching and inhibition) to deficits in real-world tasks such as walking and driving. Eye tracking techniques could inform as to whether high and low falls risk drivers differ in the way they deploy attention within a driving scene. Research should also explore other driving tasks where older adults are differentially involved in crashes, such as busy intersections [42]. The examination of on-road driving measures is also needed to validate that response time differences in the simulator translate to real-world driving. 

In summary, our results demonstrate that high falls risk older drivers respond slower than do low falls risk drivers when responding to potential dangers in a driving simulator. A multidimensional approach that includes falls risk may be useful in more accurately assessing older driver impairment.

## Figures and Tables

**Table 1:** Demographic and cognitive measures.

Measure	High falls risk	Low falls risk
	(*N* = 14)	(*N* = 14)
Age (years)	75.8 (3.3)	74.4 (5.5)
Physiological Profile Assessment score**	1.67 (.64)	.33 (.26)
Timed up and go (seconds)**	13.94 (2.6)	10.18 (2.2)
Activities balance confidence score (of 16)*	14.17 (1.4)	15.43 (.42)
Miles driven per week	55.36 (9.9)	61.42 (7.0)
Years licensed	58.86 (5.3)	57.86 (3.2)
Crashes in last 12 months	3	2
FFOV Accuracy (% Correct)	44.29 (19.9)	47.13 (18.7)
Flicker CD RT (s)	7.40 (1.13)	7.70 (1.28)
Flicker CD Accuracy (% Correct)	53.7 (10.3)	55.39 (8.4)
Computer dual-task cost (ms)*	572.7 (207.2)	354.8 (207.8)
Collisions	8	6

Data expressed as mean (SD).

*P < .05; **P < .001.

FFOV: functional field of view.

CD: change detection.

RT: response time.

Dual-task cost = dual RT − single RT.
